# A critical analysis of the global oases mapping

**DOI:** 10.7717/peerj.20508

**Published:** 2026-02-03

**Authors:** Antonio Santoro, Francesco Piras, Mauro Agnoletti

**Affiliations:** Department of Agriculture Food Environment and Forestry (DAGRI), University of Florence, Florence, Tuscany, Italy

**Keywords:** Oasis, Traditional oasis, Cultural values, Agricultural heritage, GIAHS, UNESCO

## Abstract

Oases are receiving particular attention for their capacity to provide different ecosystem services, and as examples of adaptation and resilience in the context of climate change. A 2025 study focusing on the global distribution of oases has been published by Hernández-Agüero et al. (DOI 10.7717/peerj.18884) reporting quantitative and spatial data about the global distribution of oases. An in-depth review of this global oases mapping found significantly inconsistent data with the risk of misinforming the scientific community and other relevant stakeholders about the global distribution of oases. The main critical issues are related to: (i) the oasis definition, (ii) not distinguishing between traditional and modern oases, (iii) methodological inconsistencies in the performed spatial analysis, and inadequate spatial resolution. While most common definitions of oases agree regarding the key role of (traditional) agricultural activities and practices for the oases’ origin, shaping, and preservation, the Hernández-Agüero et al. global oases mapping includes among the oases large portions of areas only characterized by natural vegetation of different types in arid areas. In addition, the global oases mapping includes intensive and, in the long term, unsustainable cultivations, and even urban areas. Traditional oases should be considered separately from intensive cultivation systems in arid areas, as only traditional oases are strictly related to high levels of agrobiodiversity, cultural values, traditional ecological knowledge, cultural landscapes, and sustainability. Oases, especially traditional ones, are characterized by different and complex characteristics with local high variability in terms of cultivated varieties, vertical and horizontal structure, and complexity of the landscape mosaic structure. An inaccurate mapping, together with the absence of a differentiation between modern and traditional oases, can lead to a misallocation of conservation resources or to misguided policy strategies. More research and training of automatic classification at a local/national level are needed due to the local/regional pattern variability, while the development of a shared oasis definition is essential for studies related to their mapping and knowledge, as well as for an effective strategy for the protection of these agroecosystems with important ecological and cultural values.

## Introduction

Hernández-Agüero et al. (2025) recently published an article on the global distribution of oases, reporting quantitative and spatial data about their global distribution, as well as a focus on the Asia and North Africa (ANA) region. An in-depth review of the published results and of the overall applied approach found significantly inconsistent data and a lack of a solid and accurate theoretical approach. This article intends to highlight these errors to stimulate a discussion about the importance of providing accurate and validated data on the presence of such biocultural heritage.

Oases are unique forms of adaptation to extreme environmental conditions characterized by water scarcity and high temperatures. These systems are excellent examples of adaptation to conditions of high aridity and, therefore, have assumed a fundamental role in recent years in the context of climate change, progressive water scarcity, prolonged drought periods, and increases in average and maximum temperatures (Leal Filho et al., 2024). In addition, oases proved to be multifunctional systems capable of providing multiple ecosystem services, *i.e.,* the benefits people obtain from ecosystems and agroecosystems (Reid, 2005) to rural communities, actively contributing to their wellbeing and livelihood (Santoro, 2023).

This growing interest in oases and the related multifunctional benefits is also recognized at the institutional level. The UN’s largest initiative dedicated to agricultural heritage, the GIAHS (Globally Important Agricultural Heritage Systems) Programme managed by the Food and Agriculture Organization (FAO), comprises different oases in five Middle East and Northern Africa (MENA) countries. This FAO Programme aims to identify and preserve traditional agro-silvo-pastoral systems that have resulted from rural communities adapting to their surrounding environments. These systems have an ancient origin but are still crucial for the well-being of rural communities as they provide multiple products and ecosystem services, thanks to their agrobiodiversity, traditional knowledge, traditional landscapes, and cultural and social values (Altieri & Koohafkan, 2004; Koohafkan & Altieri, 2011; Agnoletti et al., 2023; Agnoletti & Santoro, 2022). Oases in the MENA region are also part of the UNESCO World Heritage List, with three oases-related sites, and of the UNESCO Intangible Cultural Heritage. “Date palm, knowledge, skills, traditions and practices”, whose application has been promoted by 15 different countries, has been inscribed in the list in 2022, testifying to the importance of the date palms growing “in oases in desert areas with suitable water levels for irrigation” for the local cultural heritage dating back centuries (UNESCO, 2022). The inclusion of different oases in these international programs testifies to the global relevance of these agroecosystems and the need to develop a global database to promote knowledge among researchers, international, national, and local institutions, as well as among farmers and technicians, to develop targeted protection and valorization actions based on accurate spatial data.

In the following sections, we will detail the main critical points of the adopted approach that led to some incorrect or incomplete results.

### The issue of the oasis definition

The first issue is related to the definition of oasis. Hernández-Agüero et al. (2025) define oases as “Azonal, highly productive areas, with permanent dense vegetation and mostly converted to agriculture, embedded in drylands and with a certain degree of isolation; they facilitate high biocultural diversity and allow biological and cultural exchange through otherwise impermeable regions”. This definition is based on previous and reported literature, and even if it states that most of the oasis surface has to be “converted to agriculture”, the results of the global mapping include large areas of only natural “permanent dense vegetation”. Associating oasis with natural vegetation risk to neglect the cultural origin and values related to oases. Despite a shared or “official” definition of oasis is not available, previous literature clarifies that oases are places shaped by agricultural activities. In 1996, Battesti (1996) reported that “oases are intensive production systems of great complexity and always in a fragile balance: they cannot exist without humans”. The same author explained that oases can be considered models of extremely efficient production systems in desert areas, only relying on scarce water sources. More recently, De Haas et al. (2001) defined oases as “agricultural areas in arid environments”.

Furthermore, some of the references cited by Hernández-Agüero et al. (2025) to develop their own definition highlight the role of human activities in shaping oases. Liu, Wang & Xin (2022) reported that oases are “heterogeneous ecological landscapes” and that oases “encompass” (not correspond to) the natural environment, with human activities playing a key role. Similarly, Chen et al. (2022) define oasis as “an area with flat terrain, stable water supply, and high productivity that can support human activities such as agriculture and animal husbandry”, while according to Sraïri & Ouidat (2022), they are “areas with intense farming activities located in very arid environments”. Jia et al. (2004), instead, define oases as a “specific landscape that exists with deserts in arid regions” and consider oases as “the most concentrated area of human activities in arid regions”. Finally, according to Cui et al. (2024), oases are “important geographic units formed on the desert matrix of drylands due to the driving force of stable water sources” that result to be crucial for “local production and livelihoods” representing “the core of the human–environmental system in drylands”.

Recently, two important definitions of oases have been published, which are worth analyzing. Considering the institutional level, according to the FAO (2024), “oases are human-crafted cultivated havens nestled within vast arid or desert areas” and can be considered as cultural landscapes resulting from “ingenious human creation”, representing an “extraordinary model of harmonious relationship between man, culture, land and the environment”. Another relevant oasis definition can be found in the recent “Errachidia declaration and guidelines for the sustainable development of oasis ecosystems” (Kabiri et al., 2023), according to which “an oasis is a strategically positioned human settlement in arid geographical conditions, where local natural resources are exploited through carefully selected traditional technologies over an extended process of experiential knowledge”. A key feature of this declaration is the reference to the exploitation of natural resources according to selected traditional technologies. On one side, this strengthens the fact that oases are indissolubly linked to traditional knowledge and cultural practices; on the other side, it clarifies that such systems must be somehow sustainable in terms of the exploitation of natural resources. This is particularly important given that these ecosystems are found in arid environments, where water is the main limiting factor; we will later see how the theme of water sustainability is addressed differently depending on the type of oasis. The same declaration recognizes the multifunctional role of oases, as well as their importance for biodiversity and sustainable development, but places cultural factors, particularly traditional agricultural activity, at the backbone. The FAO and the Errachidia declarations place oases within the group of cultural landscapes (Sauer, 1925; Antrop, 1997). According to these definitions, oases do not correspond to natural vegetation growing in arid places, and that is why they can be so important for agrobiodiversity and cultural heritage. Considering the above, it would be important to consider as oases only the places characterized by the combination of human settlements and cultivated areas developed as a result of adaptation to the surrounding environment and to scarce water availability. However, as we will see later, this distinction between places characterized only by natural vegetation and places characterized by the combined presence of agricultural activity and settlements in arid areas is far from sufficient when it comes to addressing biocultural diversity and sustainability. These two different environments (cultivated oases *vs* natural vegetation) have completely different characteristics in terms of cultural importance, agro and biodiversity conservation, contribution to food security, *etc*.

**Figure 1:**
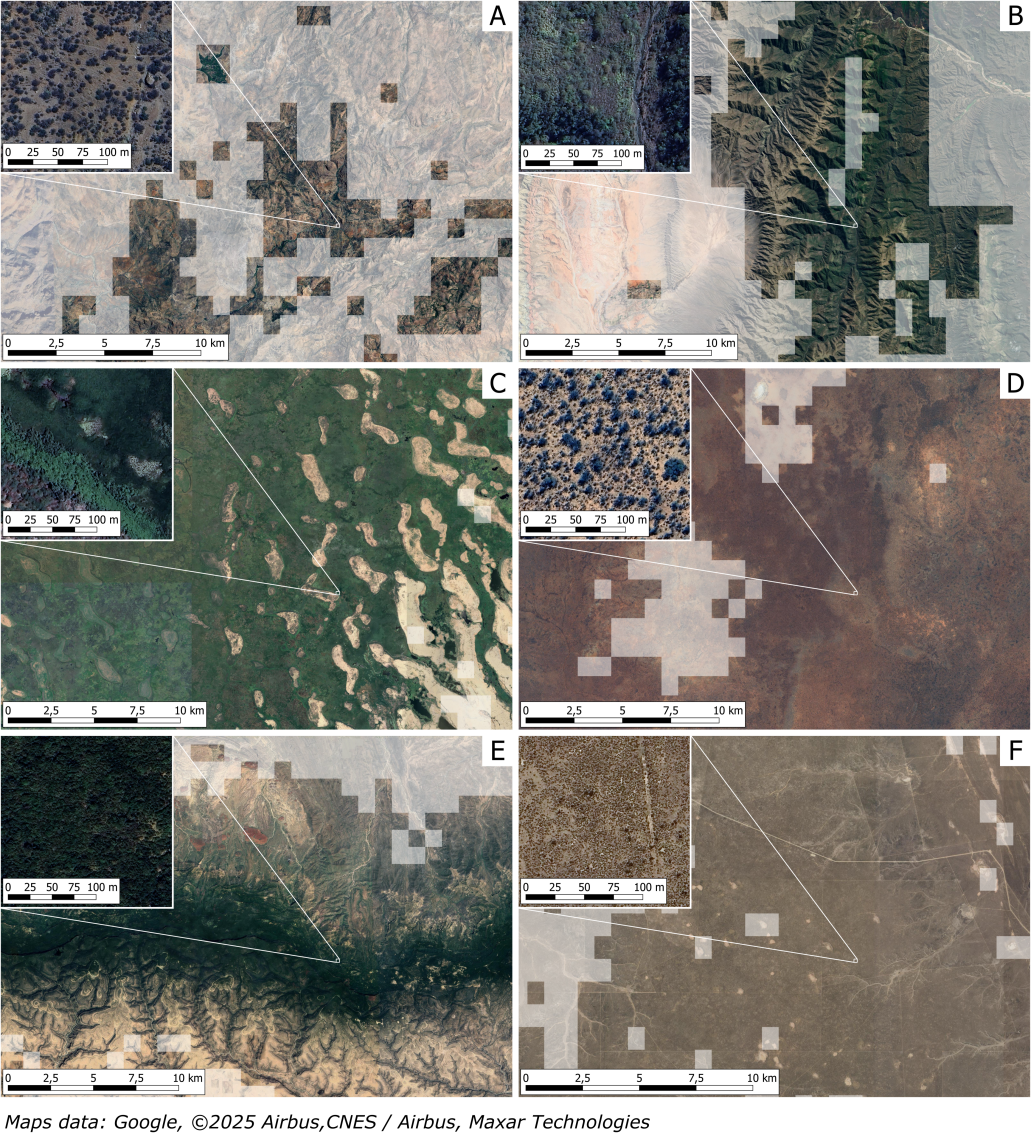
Misclassification: natural areas without agricultural activity are classified as oases. The images are related to the classification of different natural and seminatural ecosystems with no agricultural activities, as oases; the grey areas are outside the oases mapping. These examples, discussed in the text, are related to the argan forest of Morocco (A), to Andean mountain forests in Argentina (B), to Lake Chad marshlands in Central Africa (C), to Southern Australia bushland (D), to Karkaar Mountains in Somalia (E), and to shrubland in Valdes Peninsula in Argentina (F).

Many areas included in the global mapping simply correspond to natural vegetation and not to cultivated areas; therefore, they are not relevant for food security or cultural diversity, even if they can be particularly important for (natural) biodiversity, and cannot be considered places of “pivotal biocultural relevance”. If oases are considered by the authors of the global mapping places of “pivotal biocultural relevance”, the simple presence of natural vegetation in arid areas does not have these characteristics. Biocultural diversity is, in fact, “the diversity of life in all of its manifestations: biological, cultural, and linguistic, which are interrelated (and likely coevolved) within a complex socio-ecological adaptive system” (Maffi & Woodley, 2012). In addition, it is important to highlight that the presence of natural vegetation in arid areas can also be tied to the availability of water sources, but can be present thanks to specific adaptations of some vegetal species to aridity. Some examples related to large surfaces of different types of natural vegetation in arid areas erroneously considered among oases are reported in Fig. 1 and discussed below.

The argan natural forest of Morocco has been almost entirely included in the global oases mapping (Fig. 1A), even if this area is occupied by a natural low-density forest made of argan trees (*Sideroxylon spinosum*, formerly known as *Argania spinosa*). While some small oases, as Tizgui oasis (de Haas, 2006), are present within the mapped area, most of the mapped surface is only occupied by the argan natural forest, whose presence and origin is not related to human activities or to the presence of specific water sources (*wadis*, springs, groundwater, …), but depend on the adaptation of the species to arid environmental conditions (Afi et al., 2024). Local populations have the right to collect and sell nuts for argan oil production, but the trees are not planted, pruned, or managed. Even if we consider oases simply as places of natural vegetation associated with “stable water sources” (Cui et al., 2024), the argan forest can’t be considered an oasis.

The previous one is not the only case related to the inclusion of mountain forests in the global oases mapping. South of Salta, Argentina, forests located along Andean Mountain slopes at altitudes of 1,700–2,200 m a.s.l. have been considered oases (Fig. 1B). Even if some small oases are present in the region (Los Nacimientos, Fiambalá, Anillaco), most of the mapped surface corresponds to the ecosystem called “Bolivian-Tucuman Subhumid Montane Forests”, a dense to semi-dense forest with a canopy of 20–25 m in height and several understory strata and woody lianas; common species include *Parapiptadenia excelsa*, *Tipuana tipu*, *Cupania vernalis*, *Diatenopteryx sorbifolia*, *Acacia polyphylla*, *Anadenanthera macrocarpa*, *Erythrina falcata*, *Terminalia triflora* (Josse et al., 2003).

Another example of natural vegetation mistaken for oases is related to Lake Chad (Fig. 1C), a big shallow lake located at the junction of Nigeria, Niger, Chad, and Cameroon. This lake suffered a significant shrinking of the water surface in the last decades due to reduced inflow from tributaries and to variation in rainfall patterns; most of the area once occupied by the lake is nowadays occupied by marshlands (Olowoyeye & Kanwar, 2023; Gbetkom et al., 2023), with only a small part currently covered by freshwater. Although almost the entire Lake Chad area is included in the global oases mapping, the place is not characterized by cultivated surfaces, but only by marshlands, although different small oases can be found in Southern Niger (Jahiel, 1998).

A large part of Southern Australia has also been considered an oasis (Fig. 1D), while it corresponds to natural vegetation made of scattered trees and shrubs adapted to the arid climate (Groves, 1994) with no agricultural activities. In particular, most of the areas considered oases correspond to areas classified by the National Vegetation Information System as “Acacia Forests and Woodlands”, “Acacia Shrublands”, or “Mallee Woodlands and Shrublands”. According to the type, these formations vary between natural vegetation of acacia or eucalypt species, usually with canopy cover less than 30%, and by hummock grasses (spinifex), chenopods, or other woody shrubs (Australian Government Department of Climate Change, Energy, the Environment and Water, 2020).

Other large areas occupied by natural vegetation have been considered as oases, for example in north-eastern Somalia (Fig. 1E); this area correspond to the Karkaar Mountains, whose elevation is around 1,800–2,100 m a.s.l. and whose vegetation is made of scattered trees and shrubs adapted to this arid mountain climate, without the presence of significant agricultural surfaces (Boitt, Langat & Kapoi, 2018).

The last example refers to Argentina, where the Valdes Peninsula (Fig. 1F) has been entirely classified as an oasis; this area corresponds to a large natural reserve with no cultivation, whose sparse vegetation is only made up of different types of natural shrubs and herbaceous vegetation (Bertiller et al., 2017), locally called *matorral*. According to the Terrestrial Ecosystems (Reference Classification) of Argentina, developed by The Nature Conservancy corresponds to the “Atlantic Shrub Steppe Ecosystem of the Valdez Peninsula”, characterized mainly by *Chuquiraga avellanedae*, alternated with psammophilous grasslands established on the dune systems and with *Chuquiraga hystrix* in depressions and coastal areas (Roig, 1998).

**Figure 2:**
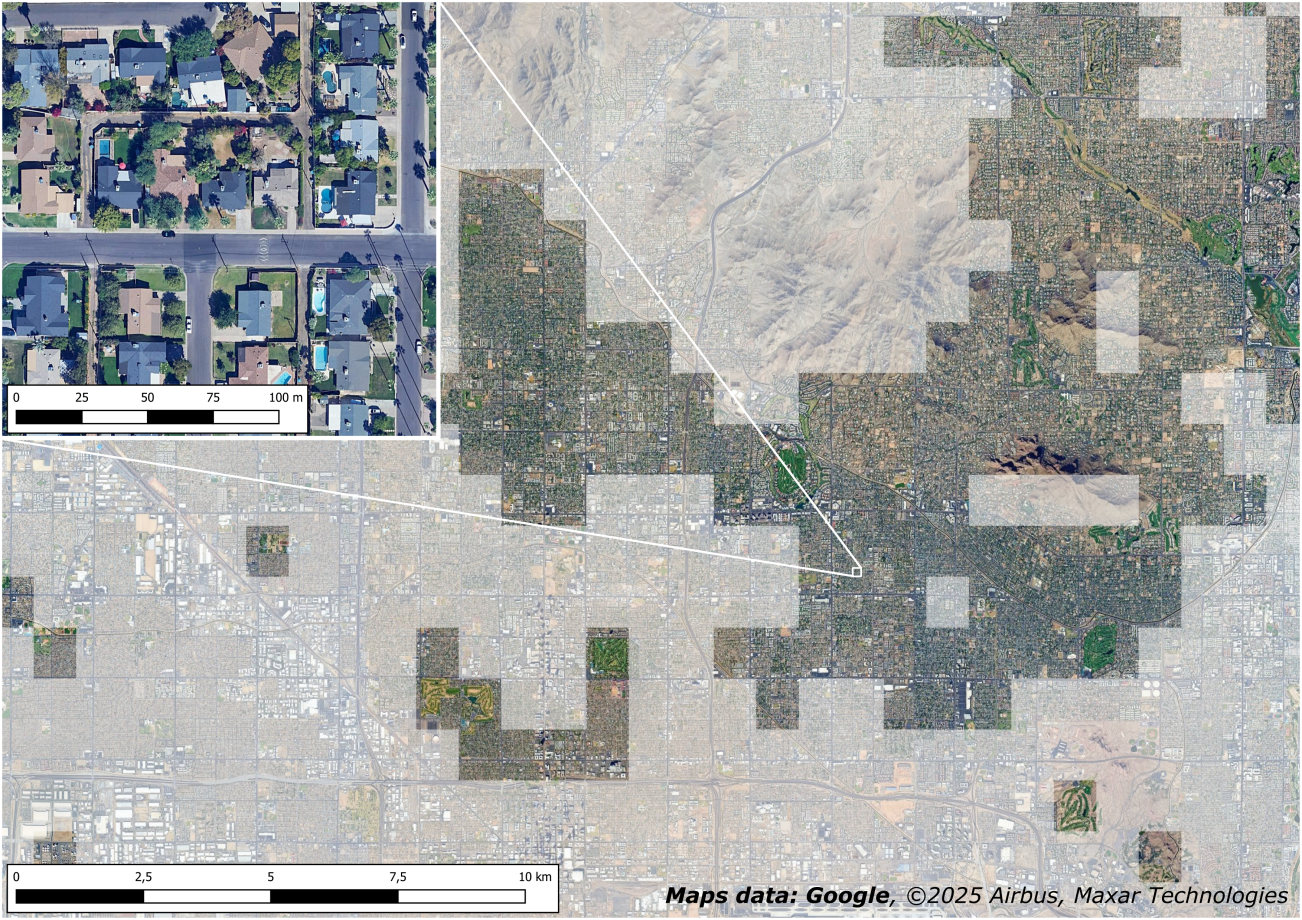
Misclassification: urban green spaces are identified as oases. Large parts of Phoenix (Arizona, USA) suburbs have been erroneously considered oases due to the presence of urban green spaces and to the arid climate.

Besides natural vegetation, portions of urban areas with relevant urban vegetation have also been included in the global mapping. It is the case of Phoenix (USA), where large parts of the suburbs characterized by urban parks, private gardens, and golf courses, have been erroneously considered oases (Fig. 2).

### Traditional oases *vs* modern oases

The second critical issue is related to the fact that no differences between traditional oases and modern intensive systems have been made, even if these two agroecosystems largely differ in origin, structure, sustainability, agrobiodiversity, and biocultural diversity. Despite significant variations that can be found at the local level, oases can normally be classified into two big groups: traditional oases and modern oases (Benaoun, Elbakkey & Ferchichi, 2014; Santoro et al., 2020). Traditional oases are often based on a three-layer vertical structure to take advantage of the so-called agroforestry effect (Nair, 1993) to cultivate different species, with the upper layer that protects the lower ones from excessive evapotranspiration. The upper layer is represented by different tree species adapted to direct sunlight, the intermediate one by other fruit trees, and the lower one is used to cultivate vegetables and fodder. In the MENA region oases the upper layer is commonly made of date palms (Santoro, 2023), while in other regions of the world or in other environments, the tree component can be represented by different tree species. In Central Asia or in China, it is common to find *Populus euphratica* as the main tree species (Wan et al., 2024), while in the cold oases of Morocco main tree species include *Populus alba* and *Populus nigra* and fruit trees (*Juglans regia, Prunus dulcis amygdalus, Ficus carica, Prunus persica, Punica granatum*). Traditional oases can also be well integrated with livestock, as they provide fodder for the raised animals, which, in exchange, produce manure for crop fertilization and a workforce for tasks such as transport, water extraction, or ploughing (Bourzat & Goe, 1990; Tisserand, 1990). Traditional oases do not follow a regular planting scheme as they have a high internal property fragmentation and reduced overall size (Xue et al., 2022), with agricultural operations mainly conducted manually and lower yields (Ministère de l’environnement et du développement durable, 2015; Lasram, 1990). Finally, traditional oases usually also rely on a community-based water management system that works on the basis of traditional local rules and sometimes foresee collectivized land commonly used as pasture at the border of the oases (El Jamali, 2011).

Modern oases, instead, can be defined as simple date palms or other fruit trees plantations with a regular planting scheme, higher annual yields, a predominance or even a monoculture of the most productive and easily marketable varieties, no or very rare intercropping and no integration with animal husbandry, large property size (Lasram, 1990), and use of modern irrigation systems and intensive groundwater extraction.

While traditional oases and related traditional cultural landscapes effectively contribute to the preservation of cultural values and agrobiodiversity (Houssni et al., 2023; Peano et al., 2021), modern oases are often responsible for water sources overexploitation or salinization, and, due to the use of chemicals, they can be responsible for groundwater pollution too (Dhaouadi et al., 2021; Wang et al., 2022). Modern oases in the MENA region are one of the main responsible of waterlogging and salinization (Hachicha & Aissa, 2014; Ali & Salem, 2024). Therefore, another key difference between traditional and modern oases is related to the sustainable or unsustainable use of water resources. As demonstrated by Luedeling & Buerkert (2008), the shift from traditional to modern systems and consequent land use changes affected the hydrological sustainability of mountain oasis systems in northern Oman between 1978 and 2005. According to Raddadi & Podwojewski (2022), who performed a complete inventory of the springs in the Nefzaoua region (Tunisia), 89% of artesian springs that supported the local oases for centuries are dried out or degraded due to the intense irrigation required by recently established modern date palm plantations. The sustainable use of water sources in traditional oases has been guaranteed for centuries by a wise use of traditional irrigation systems (*qanat*, *foggara*, *khettara*) (Santoro, 2023), whose abandonment caused water-related problems in the long term as well as social contrasts (Rezzoug, 2019; Benqlilou & Bensaid, 2013). The management and knowledge related to traditional irrigation systems is only one of the different cultural ecosystem services related to traditional oases, which include knowledge system, aesthetic value, recreation and ecotourism, sense of place, and social relations (Santoro, 2023). In addition, modern intensive systems do not contribute to agrobiodiversity; instead, they can represent a significant threat. For example, date palm monocultures are replacing traditional oases in most North African countries, with the loss of traditional date varieties in favor of Deglet Nour monocultures (Addoun et al., 2023; Ben Khalfallah et al., 2021).

**Figure 3:**
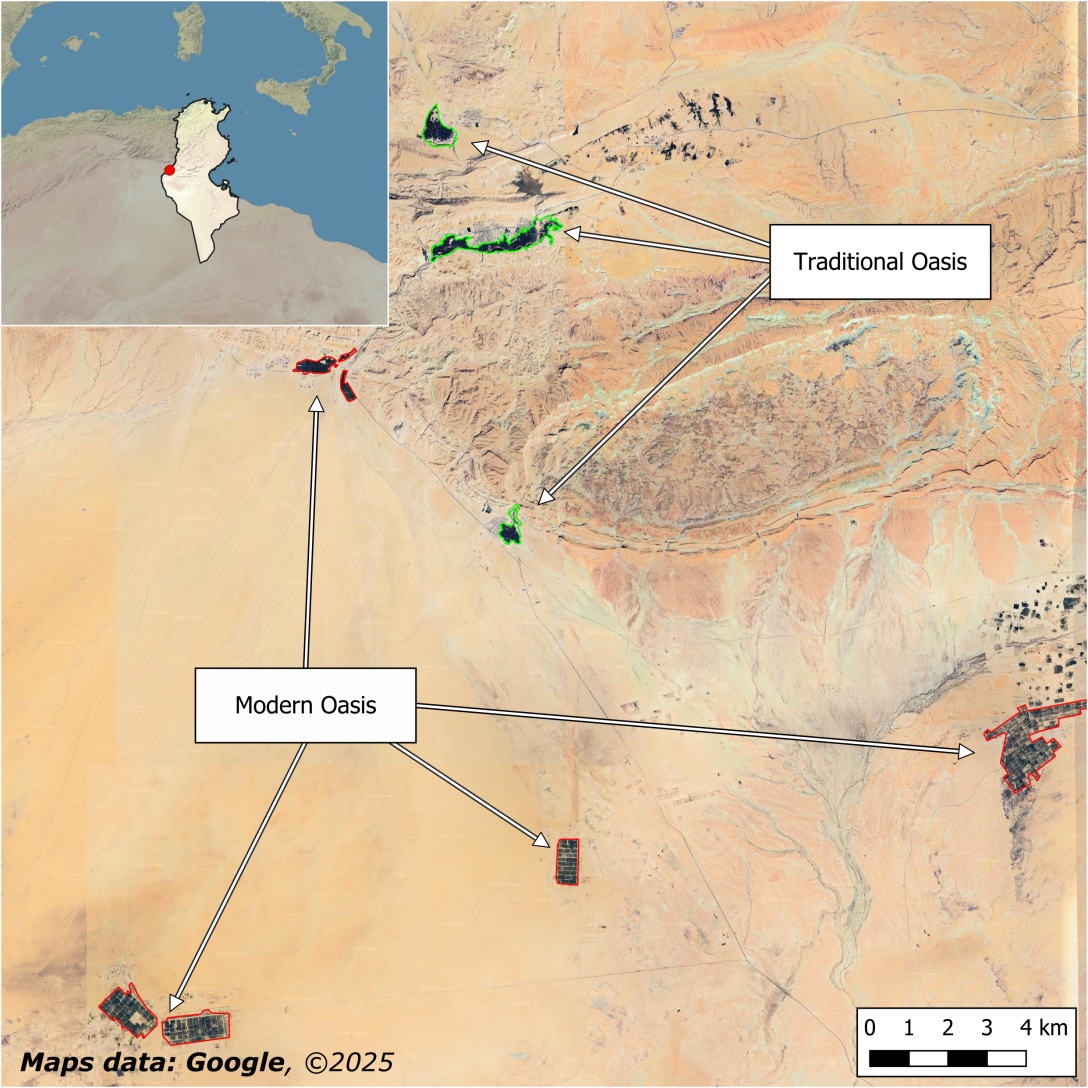
Traditional and modern oases. In some cases, as in this image referring to Tozeur Governorate in Tunisia, modern intensive oases have been mapped together with traditional oases.

In some cases, modern intensive oases have been mapped together with nearby traditional oases, as in the case of the three traditional mountain oases of Mides, Chebika, and Tamaghza (Tozeur Governorate, Tunisia) (Santoro et al., 2020) (Fig. 3). Of course, this is an intrinsic limitation of the applied methodology based on supervised automatic classification, but considering the completely different roles of traditional and modern oases for biocultural diversity and sustainability, it would be important to distinguish among them also to avoid misallocation of conservation resources and misguided policy decisions.

**Figure 4:**
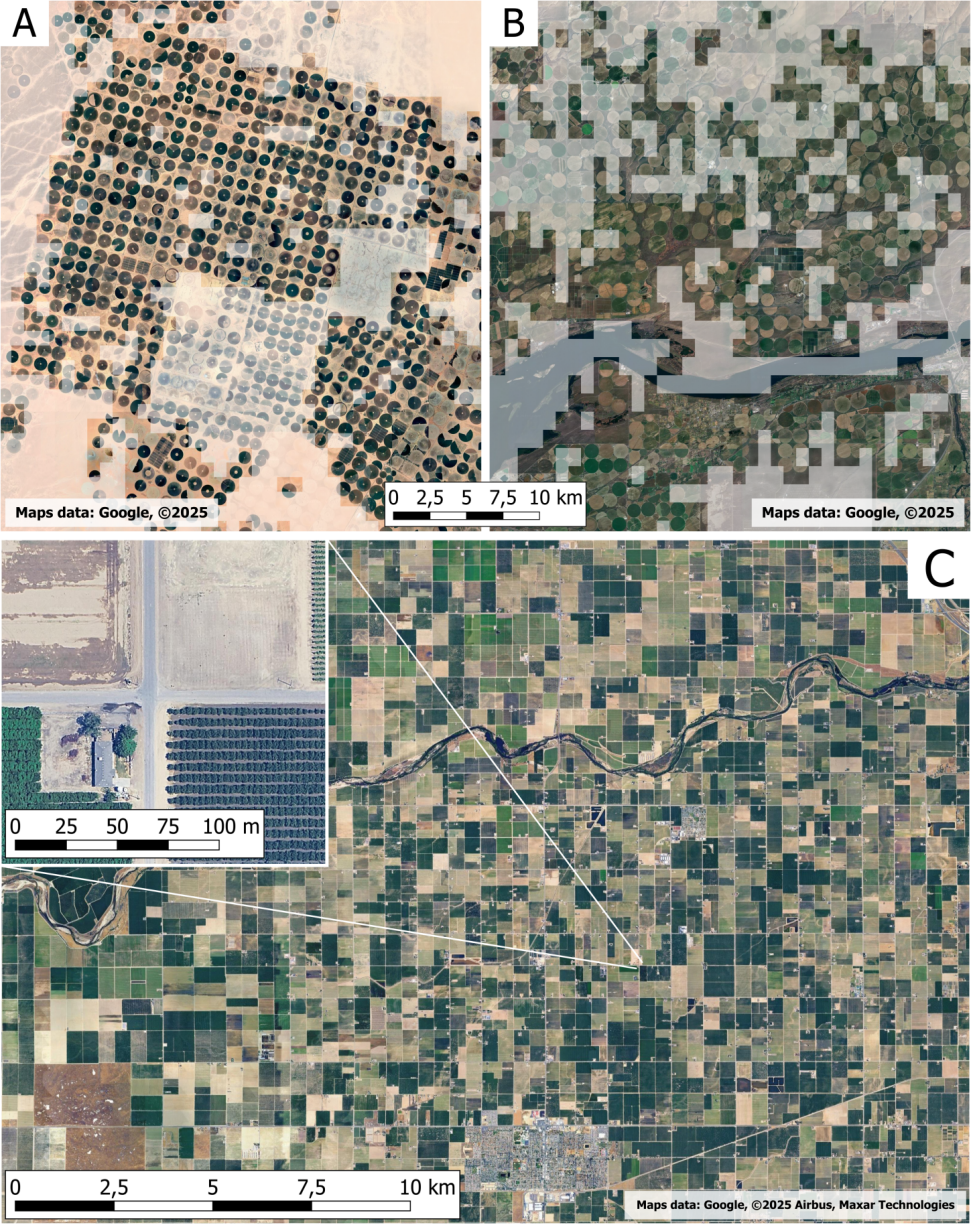
Misclassification: intensive cultivation considered as oasis. Examples of intensive center-pivot cultivations in Saudi Arabia (A) and in Oregon, USA (B). Other typologies of intensive cultivations have also been included by Hernández-Agüero et al. (2025) in the global oases mapping, like in California, USA (C).

One of the typologies of cultivated areas in arid places that have been mapped in the Hernández-Agüero et al. (2025) research corresponds to round-shaped intensive cultivations with center-pivot irrigation (Fig. 4). These intensive systems have spread in many arid areas since the 1980s due to technological development and the possibility of extracting water from greater depths, but, in the long term, proved to be unsustainable in terms of water and consumption (Brik, Guerrah & Atia, 2022) and for the use of chemicals. It has been proven that this type of cultivation can be responsible for groundwater overexploitation in desert and arid environments. *i.e.*, in the Souf region of Algeria, potato cultivations with center-pivot irrigation could consume up to 10,000 m3/ha/cycle of water for an average cycle of about 120 days, compared to 500 m3/ha/cycle in farms using drip or precision irrigation systems, being responsible for the depletion and lowering of the water table with consequent problems for the local traditional oases (Faci, Oubadi & Madi, 2025). In addition, these systems can lead to a progressive and significant increase in soil salinity, as some authors reported for Saudi Arabia (Al-Ghobari, 2011). Finally, these round-shaped cultivations are not linked at all to agrobiodiversity or/and cultural values, as they correspond to modern monocultures of non-local varieties and often turned out to be environmentally unsustainable in the long term; therefore, they can’t be considered oases, taking into consideration the biocultural diversity, the structure, or their sustainability.

Distinguishing between natural vegetation, traditional oases, and modern oases is, therefore, of crucial importance, as it is not possible to perform any inventory or mapping without defining in advance with high accuracy the object of the study. As we demonstrated, these three different (agro)ecosystems have completely different characteristics in terms of sustainability, (agro)biodiversity and genetic diversity, cultural, social, and ecological values. In the following table (Table 1), we summarize the main characteristics of these three systems.

**Table 1:** Main characteristics of traditional oases, modern oases, and natural vegetation in arid areas.

Traditional oases	Modern oases	Natural vegetation
Presence of agroforestry (combined presence of trees, herbaceous crops and fodder, or of livestock), usually organized on the basis of a three-layer vertical structure	Absence of (or very rare) agroforestry or intercropping	Absence of significant agricultural activities
High genetic diversity (agrobiodiversity): presence of various local species and varieties	Low genetic diversity (agrobiodiversity): predominance or monoculture of the most productive varieties	Medium-high genetic diversity (biodiversity)
High habitat and microhabitat diversity	Low habitat and microhabitat diversity	Medium habitat and microhabitat diversity
Integration with livestock breeding: animals provide manure and in “exchange” oases produce fodder to feed the animals	No integration with livestock breeding	No pastoral activities
Low-medium annual yields of the main cash crop	High annual yields of the main cash crop	
No regular planting scheme of the tree component	Regular planting scheme of the tree component	Natural origin of the tree component
Mainly manually conducted agricultural operations especially in case of date palms (pollination, fruits thinning, harvesting)	Mechanization of most of the agricultural operations	
Scarcity of water resources	Scarcity of water resources	Scarcity of water resources
Presence of traditional irrigation systems (foggara, qanat, khettara,…)	Presence of modern irrigation systems (irrigation networks, pumps,…)	No artificial irrigation systems
High degree of collectivized water management	No collectivized water management	
High fragmentation of the farm properties	Large farm size	
High provisioning of cultural ecosystem services (knowledge system, aesthetic value, recreation and ecotourism, sense of place, social relations)	No or very low provisioning of cultural ecosystem services (knowledge system, aesthetic value, recreation and ecotourism, sense of place, social relations)	No or very low provisioning of cultural ecosystem services (knowledge system, aesthetic value, recreation and ecotourism, sense of place, social relations)

### Methodological inconsistencies and implications

The methodology applied appears at times confused and unclear. In particular, we identified some issues that require specific attention.

First, there is a lack of clarity regarding the spatial scale used in the analyses. The article specifies the pixel dimensions for each layer (30 m for NDVI, 1,000 m for aridity, 4,638.3 m for other variables); however, the text refers to 100 m pixels for the training of the Random Forest model, and the results are subsequently presented using 1,000 m pixels. No explanation is provided regarding how values were aggregated. This makes it difficult to evaluate the final output, as the aggregation method used to upscale from 30 m to 100 m, and then to one km resolution, is not disclosed. Furthermore, upon examining the Google Earth Engine code published as Supplemental Information, the grain size used for training the Random Forest model appears to be set at 1,000 m, unlike what has been reported in the original paper (100 m).

Another critical methodological issue concerns the discrepancy between the spatial resolution of the predictor datasets used in the Random Forest (RF) model and the scale at which the model was applied. Although the authors state that the analysis was conducted at a fine resolution (30 m or 100 m), the majority of predictors originate from datasets with significantly coarser spatial resolutions, such as one km (*e.g.*, aridity index) or even coarser (up to 4.6 km for climatic variables). As a result, when the RF model is applied to 30 m cells, all pixels within a given one km cell receive identical values for nearly all predictors, with the exception of the median NDVI, which is the only variable available at a resolution compatible with the analysis.

This configuration implies that, despite the apparent spatial granularity of the output, the model lacks truly differentiated information to distinguish between adjacent pixels, except through the contribution of NDVI. In this context, one would expect the median NDVI to emerge as the dominant predictor in the classification process. However, according to the results reported by the authors, it ranks only fourth in relative importance among the ten predictors used. This finding appears contradictory and suggests that the structure of the training dataset—particularly the selection of absence points—may have distorted the predictor importance hierarchy.

Specifically, the choice of globally distributed absence points, selected solely based on high NDVI values without consideration of climatic context, likely led the model to prioritize the aridity index as the primary discriminant. A more effective strategy would have involved selecting non-oasis points within arid environments, ideally in proximity to the presence points, thereby ensuring greater ecological coherence between classes and facilitating model learning. Such an approach would have strengthened the RF’s ability to distinguish oases from other vegetated contexts typical of arid regions, such as shrublands, irrigated areas, or anthropogenic settlements, reducing the risk of misclassifying oases with ecologically dissimilar environments.

A further issue concerns the ambiguity between model accuracy and map accuracy. In the work of Hernández-Agüero et al. (2025), a 99% accuracy is reported for the Random Forest (RF) model, correctly calculated on a 20% subset of the training points reserved for validation. However, this value refers exclusively to the classifier’s performance on the training data and provides no direct indication of the accuracy of the resulting global oases map. The absence of an explicit distinction between internal model validation and independent evaluation of the cartographic product may easily lead readers to misinterpret this figure as representative of the overall map quality.

The lack of an independent assessment of map accuracy, based on a probabilistic sample representative of the spatial domain, constitutes a significant methodological gap. While it does not compromise the internal validity of the model, this aspect should at least be acknowledged in the discussion or conclusions as a limitation of the study and as a suggestion for future research aimed at evaluating the generalizability of the product at a global scale.

## Conclusions

Oases, especially traditional ones, represent multifunctional agricultural areas that have provided food security and vital ecosystem services to local communities for centuries. In the context of climate change, these cultural landscapes offer inspiring examples of sustainable agricultural practices based on the careful use of scarce water resources. Given the mounting socio-economic and environmental challenges they are facing, their protection and valorisation is of utmost importance.

To effectively safeguard these unique landscapes, it is essential to deepen our knowledge of their various characteristics—ranging from their internal structure and the functional relationships among different features, to their precise spatial location. A global mapping of oases, combined with a shared and updated definition, is therefore fundamental. While the perception of oases’ characteristics and typologies among local farmers and communities is often clear and vivid, academic discourse reveals considerable ambiguity. Indeed, a univocal definition is difficult to achieve since oases are governed by a multiplicity of factors—ecological, economic, social, political, and historical—that make them extremely diverse from one region to another. Although several definitions of oases coexist, most agree that these areas are substantially shaped by agricultural activities, thereby excluding natural vegetation in arid regions from the concept. The definition included in the “Errachidia declaration and guidelines for the sustainable development of oasis ecosystems” (Kabiri et al., 2023) currently stands as the most appropriate and updated one. A critical issue that is not addressed by this definition is related to the use of water sources. Considering that traditional oases have been managed for centuries only relying on the wise management of local scarce water sources and that one of the main threats related to the spread of modern oases is the unsustainable use of water sources in the long term, it would be crucial to include the issue of the sustainable use of water sources in the definition of oases. This is clearly related to the need of distinguish between traditional and modern oases, as modern oases have been shown to contribute to biodiversity loss, groundwater overexploitation, salinization, pollution, and overall unsustainability in the long term.

Technological tools now available in spatial analysis and remote sensing, while promising, are not yet fully suitable for a global mapping of oases. Their variable local characteristics—in terms of vertical and horizontal structure, the complexity of the landscape mosaic, the reduced size and high fragmentation of many traditional systems, and the limited accuracy of automatic classification systems—pose significant challenges. The approach by Hernández-Agüero et al. (2025), despite its interesting attempt to correlate oases’ distribution with cultural values, rests on the erroneous assumption that every type of vegetation in arid environments is indicative of an oasis. This oversimplification has led to clear classification errors and inaccurate estimates of oases’ distribution and extent, which may misinform both the scientific community and other stakeholders.

These observations suggest that to create a comprehensive global atlas of oases, it is imperative to begin with localized studies—preferably at the national or regional level—where data and methods can be finely tuned to the specific environmental, cultural, and historical contexts of each area. Moreover, leveraging existing partial censuses and local inventories can provide a solid foundation for building a more accurate and context-sensitive mapping system. By integrating high-resolution local studies with broader-scale analyses, researchers can progressively refine our understanding of oases, enhancing both their conservation and sustainable management. An inaccurate mapping, together with the absence of a differentiation between modern and traditional oases, can lead to a misallocation of conservation resources by national or regional authorities, or to misguided policy strategies and decisions based on incorrect spatial data or sustainability assumptions.

In summary, while our critique has highlighted methodological shortcomings in current global mapping efforts, it also opens up a constructive pathway forward. By embracing a bottom-up approach that respects the local diversity of these cultural landscapes and by making full use of existing data sources, the scientific community can work towards a refined, reliable, and dynamic atlas of oases—one that truly reflects their ecological, cultural, and socio-economic significance.

## Additional Information and Declarations

### Data Availability

The following information was supplied regarding data availability: Shapefile of points used for validation.
